# Relationship between parental estimate and an objective measure of child television watching

**DOI:** 10.1186/1479-5868-3-43

**Published:** 2006-11-27

**Authors:** Jodie L Robinson, Dana D Winiewicz, Janene H Fuerch, James N Roemmich, Leonard H Epstein

**Affiliations:** 1 Department of Pediatrics, School of Medicine and Biomedical Sciences, State University of New York, Farber Hall; Room G56; 3435 Main Street; Buffalo, NY 14214, USA

## Abstract

Many young children have televisions in their bedrooms, which may influence the relationship between parental estimate and objective measures of child television usage/week. Parental estimates of child television time of eighty 4–7 year old children (6.0 ± 1.2 years) at the 75^th ^BMI percentile or greater (90.8 ± 6.8 BMI percentile) were compared to an objective measure of television time obtained from TV Allowance™ devices attached to every television in the home over a three week period. Results showed that parents overestimate their child's television time compared to an objective measure when no television is present in the bedroom by 4 hours/week (25.4 ± 11.5 vs. 21.4 ± 9.1) in comparison to underestimating television time by over 3 hours/week (26.5 ± 17.2 vs. 29.8 ± 14.4) when the child has a television in their bedroom (p = 0.02). Children with a television in their bedroom spend more objectively measured hours in television time than children without a television in their bedroom (29.8 ± 14.2 versus 21.4 ± 9.1, p = 0.003). Research on child television watching should take into account television watching in bedrooms, since it may not be adequately assessed by parental estimates.

## Findings

Young children today are exposed to an abundance of sedentary activities that include television and movie watching, using the computer, and playing video games. National data and recent research both suggest that 3–7 year-old children watch an average of two [1-7] to three hours of television per day [2-9], with media related television activities such as video games and movies adding a half hour per day [6,10], and computer games adding another half hour per day [6,11].

Television viewing is typically assessed by parental estimates, since young children cannot accurately self-report their own television viewing behaviors, but there is limited validity data of parental estimates of child television viewing time. Parents overestimated child television viewing by 1.1 hours/week to 5.3 hours/week in comparison to parental diaries [12], and parental diaries overestimated television viewing when compared with video observation by 3.2 hours/week [12]. Parents also underestimate child viewing hours versus Nielsen ratings (13.4 hours/week versus 27.8 hours/week, respectively) [9]. Nielsen Media Research measures estimates of audience viewing using an electronic monitoring system, called people meters, placed on each TV set in randomly selected homes. The people meters record what channel is being watched, and who is watching to provide national household and person estimates of TV viewing [9]. Parental estimates are influenced by how much opportunity parents have to observe their children engaging in these behaviors. Thirty-two to forty percent of children seven years old and younger have a television in their bedroom [6,10,13], which may limit the accuracy of parental observations on television watching. A television set in the child's bedroom is related to increased prevalence of obesity [13], higher overall viewing times [11,13-16], and greater sleep disturbances [15]. This study compared parental estimates of television and computer use with an objective measure for all devices in the home, and assessed whether having a television in the bedroom influenced these estimates. Families were recruited for a study that evaluated the effects of modifying the home television watching environment on BMI change. Eligibility criteria included 1) having a 4–7 year old child at the 75^th ^BMI percentile or above, 2) having at least one working television in the home, and 3) a minimum of 14 hours per week of television and computer usage by the 4–7 year old child initially assessed by parental estimate during the phone screen, and confirmed by baseline TV Allowance™ measures. Parental estimates and objective measures of television and computer hours per week are from the baseline data of 80 families. The participating parent completed a questionnaire which assessed the number of televisions, television video game units, VCR videos/DVDs, and computers in the home.

Approximately one week later, a research assistant went to each family's home to attach a TV Allowance™ (Mindmaster Inc., Miami, FL) device to each television and computer monitor. The TV Allowance™ is an automated device that controls and monitors the use of televisions, television-based sedentary activities (including video game systems, DVD players, and VCRs), and computers. The television or computer monitor power cord plugs into the TV Allowance™, the plug is locked in, and the device is then plugged into the wall. To turn on the television or computer monitor, each family member used an individually selected four digit code. The TV Allowance™ cumulates the use of each device for each code to objectively determine use of that device by each different family member. Each family is instructed that the target child's code must be entered into the TV Allowance™ when the child is watching TV simultaneously with other family members. Baseline television and computer usage were measured over a three week period, obtained by recording the grand total reading from each TV Allowance™ device and dividing by 3 weeks. The research assistant recorded the number of televisions and computers and their locations in the home, including if a television was present in the bedroom of the targeted child. The two primary dependent measures were parental estimate of the usual number of hours per week that their child spent engaging in each behavior in the home and the objective measure obtained by the TV Allowance™. Since 11 families did not have computers, and only 2 families had a computer in the child's bedroom, analyses are reported for television time only. Linear contrasts were used to compare means for significant interactions, and ICC's were computed to assess the agreement between the two measures. Table 1 shows the demographics of the study sample by whether or not the child had a TV in the bedroom. Almost one-quarter (23.8%) of the families had a television in the bedroom of the participating child.

**Table 1:** Descriptive characteristics of participating 4–7 year old children and households.

	Total (N = 80)	TV in Bedroom (N = 19)	No TV in Bedroom (N = 61)
Variable			
Gender (boys/girls)	42/38	10/9	32/29
Demographics	Mean ± SD	Mean ± SD	Mean ± SD
Age (years)	6.0 ± 1.2	6.4 ± 1.0	5.9 ± 1.3
BMI (kg/m^2^)	19.2 ± 3.0	20.1 ± 4.4	19.0 ± 2.4
BMI percentile	90.8 ± 6.8	91.1 ± 6.7	90.7 ± 6.9
zBMI (standardized measure of BMI)	1.6 ± 0.6	1.6 ± 0.6	1.6 ± 0.6
Environment			
Televisions in the home	3.0 ± 1.3	4.1 ± 1.6	2.6 ± 1.0†
Computers in the home	1.0 ± 0.6	1.0 ± 0.6	1.1 ± 0.6
People residing in the home	4.3 ± 0.9	4.4 ± 1.0	4.2 ± 0.9
Parental estimates of television (hours/week)	25.6 ± 13.0	26.5 ± 17.2	25.4 ± 11.5
Actual television (hours/week)	23.4 ± 11.1	29.8 ± 14.4	21.4 ± 9.1‡
Ethnicity	N (%)	N (%)	N (%)
White	62 (77.5)	15 (78.9)	47 (72.1)
African American	4 (5.0)	1 (5.3)	3 (4.9)
Hispanic	7 (8.8)	2 (6.3)	5 (8.2)
More than one race	7 (8.8)	1 (5.3)	6 (9.8)

Note: zBMI was calculated using the National Center for Health Statistics growth charts from the Centers for Disease Control and Prevention (CDC) [17]

† *P *< 0.001

‡ *P *< 0.005

Children with a television in their bedroom spent an additional 8 and a half more hours per week of television than those without a television in their bedroom (29.8 ± 14.2 vs. 21.4 ± 9.1), determined by the objective measure, F(1,78) = 9.15, p = 0.003. There was no significant difference between objectively measured television hours per week for minority versus non-minority children (p > 0.05). Families that had a television in the child's bedroom had significantly more televisions in their house (4.1 ± 1.6 vs. 2.6 ± 1.0), F(1,78) = 24.60, p < 0.001.

There were no differences between parent estimated (25.6 ± 13.0) and objectively measured (23.4 ± 11.1) television time per week (F(1,78) = 0.04, p > 0.05) when television in the bedroom was not considered. The number of televisions in the house was positively correlated with parental estimates (r = 0.23, p < 0.05), but not with actual viewing time (r = 0.09, p > 0.05). A significant correlation was observed between parental estimates and television hours recorded by the TV Allowance™ (r = 0.49, p < 0.001). However, intraclass correlation coefficients showed poor agreement between parental estimates and the objective measure of television time for all subjects, those with a television in the bedroom, and those without a television in the bedroom (ICC = 0.48, 0.47, 0.47, respectively; an ICC of 0.8 or greater is considered a strong correlation), strengthening the argument that parents are inaccurate reporters of their child's television use.

**Figure 1:**
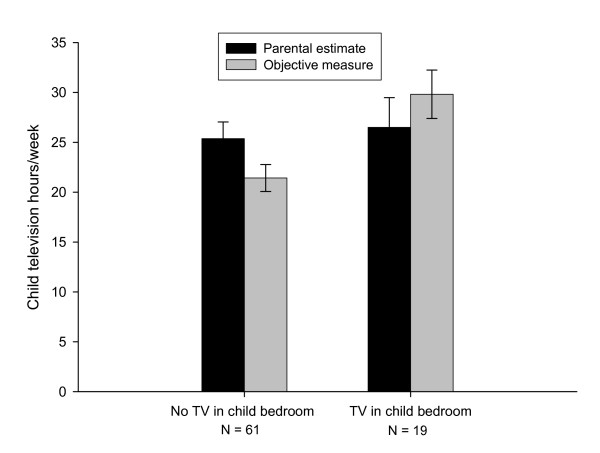
**Parental estimate and objective measure of children without a bedroom television compared to children with a bedroom television**. Parents overestimate their child's television hours/week when no television is present in the child's bedroom, while they underestimate their child's hours when the child has a television in the bedroom (p = 0.02).

Mixed analysis of variance showed an interaction between television in the bedroom and parental estimates versus objectively measured television time (F(1,78) = 5.44, p = 0.02), and the effect remains when controlling for the number of televisions in the house (F(1,77) = 13.58, p < 0.0001). As shown in Figure 1, parents overestimated their child's overall television hours when there was not a television in the bedroom (25.4 ± 11.5 hours/week versus the objective measure of 21.4 ± 9.1 hours/week), but underestimated child television watching when a television was present in the participating child's bedroom (26.5 ± 17.2 hours/week versus 29.8 ± 14.4 hours/week). While parents overestimate their children's viewing time by about 3.1 hours/week, which is similar to the 3.2 hours/week overestimation that was found between parental diaries and time-lapse video [12], they underestimate television watching when there is a television in the child's bedroom in comparison to overestimation when there is not a television in the bedroom. It may be challenging for parents to accurately monitor all television and computer use if the television or computer is not in a family space. It is possible that many parents are sensitized to television watching because of the volume of media reports about excess television watching in children, and parents thus may tend to overestimate this behavior. However, parents who place a television in a young child's bedroom may be less sensitized to media reports of excess television use. Parental overestimation of child television time when no television was present in the child room may be related to studying 4–7 year-old children at or above the 75^th ^BMI percentile. Perhaps parents attribute their child's weight status to excessive television use.

The differential results may also be related to collection of parental estimates prior to collection of objective measures. The time frame used by parents to estimate "usual" television watching may have been different than the time frame of the objectively measured television and computer use. These results were collected at baseline, and it is unknown whether the under or overestimation observed at baseline is consistent over time. If objective measures of television and computer use are not available, research is needed to evaluate ways to improve the sensitivity of parents to the amount of television and computer time their young children engage in.

## Competing interests

The author(s) declare that they have no competing interests.

## Authors' contributions

JLR drafted the manuscript and coordinated and participated in the study design and analysis. DDW helped to draft the manuscript, participated in study implementation, and performed the statistical analysis under the advisement of LHE. JHF participated in study implementation and manuscript writing. JNR participated in the design of the study and manuscript. LHE developed and directed the overall study, coordinated the design, and developed an analysis plan. All authors read and approved the final manuscript.
